# Positive risk-taking within social care for adults with physical disabilities: A review of guidelines in practice in England

**DOI:** 10.1177/03080226241246511

**Published:** 2024-04-15

**Authors:** Leanne Field, Liana Nagy, Tarnya Knaggs, Johnny Collett

**Affiliations:** 1 Centre for Movement, Occupational and Rehabilitation Sciences, Oxford Brookes University, Oxford, UK; 2 Adult Social Care, Wokingham Borough Council, Berkshire, UK; 3 College of Health, Medicine and Life Sciences, Brunel University London, Uxbridge, UK

**Keywords:** Positive risk-taking, Adult Social Care, physical disabilities, risk enablement, risk assessment, therapeutic risk

## Abstract

**Introduction:**

Despite practice guidelines, professionals do not feel confident in implementing positive risk-taking. This may be due to the guidance provided.

**Method:**

A scoping review of current organisational guidance for the professional practice of positive risk-taking within Adult Social Care services for people with a physical disability. Guidelines were obtained from Local Authorities in England in October 2020. The data were extracted using TIDieR to describe positive risk-taking as an intervention. The quality of the guidelines was assessed using AGREE II.

**Findings:**

In all, 36 Local Authorities responded out of 106 contacted. A total of 21 documents were included for review. Substantial variability was found in terminology, definitions and risk grading between documents. The greatest consistency was found in how to implement a positive risk-taking intervention. Consistency was also found in the policy that documents cited. There was little reference to evidence to support intervention components. Overall, AGREE II quality scores were low and stakeholder involvement, specifically with regard to the views and preferences of service users, was largely absent.

**Conclusion:**

There is a need for a greater consensus to guide the professional practice of positive risk-taking. Determining the extent of current evidence and establishing an evidence base may facilitate more consistent guidelines and support professionals’ confidence in implementing positive risk-taking.

## Introduction

Risk assessments in Adult Social Care (ASC) are used to mitigate the likelihood or severity of harm occurring to individuals, who may be otherwise unable to protect themselves due to disability, illness or injury (Taylor, 2022). Risk aversion is an inherent behaviour in humans and is somewhat reinforced in professional cultures for fear of being reprimanded, particularly in malpractice claims (Atwal et al., 2011). Bailey et al. (2013) found that ‘*concern with litigation undermines positive responses to risk*’ and therefore, professionals can be risk averse owing to their duty of care to avoid harm to an individual and wide belief that harm is unacceptable.

Risk is a ubiquitous aspect of daily living, and choices are constantly being made by weighing the potential for positive outcomes against the potential for harm. Indeed, the term ‘risk’ in itself has little meaning without an adjective (in other words, ‘high risk’ or ‘low risk’), as risk is merely the likelihood of an outcome occurring. Furthermore, the value individuals put on outcomes is personal and subjective, and as such, choices that others may deem unwise may be ‘worth the risk’ to the individual.

It is every individual’s right to make an unwise decision and ‘*risk is no longer an excuse to limit a person’s freedom*’ (Veselinova, 2014). To support client-centred practice, organisational guidelines have been developed for what has commonly been termed ‘Positive Risk Taking’ (PRT; Royal College of Occupational Therapists (RCOT), 2018) – whereby the risk taken is not necessarily seen as a positive choice, but the outcome may be.

While the practice of PRT has been widely encouraged within both health and social care settings to promote participation in volitional choices (Blood and Wardle, 2018), it is understandable that fear of engaging with PRT, where there may be resulting harm to the individual, might be a barrier to professionals. Indeed, it has been identified that professionals do not feel confident in doing so (Reddington, 2017) and there needs to be an occupational focus on how risk is embraced to evoke successful risk management skills (Newman et al., 2022a).

Organisational guidelines are intended to prompt a professional’s decision-making process (RCOT, 2018) and should provide a structured framework so that practitioners can be confident in their decision-making. However, many professionals say they are nervous about a punitive culture and that the guidelines available for PRT are confusing and open to interpretation without tangible examples of positive risk-taking (Seale et al., 2013).

In the UK, the RCOT produced guidelines for Occupational Therapists on PRT in 2018. RCOT asks professionals to embrace risk and work in a client-centred way. The guidelines provide an example of *‘the grandmother who wanted to go home but was kept in hospital because her cluttered home was perceived as a risk’* – pointing out that this approach restricts an individual from achieving their full potential and increases the risk of ill health. However, the extent to which these guidelines have been adopted is unknown. Furthermore, it is unclear what organisational guidelines are available for professionals practising PRT for adults with physical disabilities in England, and if there is consistency in guidelines towards PRT within ASC. Therefore, we sought to identify and evaluate current organisational guidelines and evaluate the content, consistencies, conflicts and/or differences.

## Method

### Design

We utilised scoping review methodology to map current guidelines. This methodology was chosen as the principles of scoping reviews are consistent with our aims to identify knowledge gaps and clarify concepts. Therefore, we used the Preferred Reporting Items for Systematic Reviews and Meta-analysis Extension for Scoping Reviews (PRISMA-ScR) to guide our reporting (Tricco et al., 2018). The Population, Concept and Context framework was used as a recommended approach for scoping reviews (Pollock et al., 2023) and applied to our objectives of identifying and collating organisational guidelines for PRT for adults with physical disabilities.

### Information sources and search

We contacted Local Authorities in England that provide an ASC service. LF requested copies of their documents relating to PRT using contact details from Local Authority websites. Where Local Authorities referred to another source that they used to provide their PRT guidance, this was retrieved.

### Eligibility criteria

*Population*: Adults aged 18 and above, with physical disabilities in England. Documents solely designed for use within mental health practice were excluded.

*Concept*: PRT guidelines; a broad definition of the term ‘guideline’ was applied to include definitions such as ‘tool’, ‘framework’ and ‘policy’. There were no date restrictions regarding when the documents were developed.

*Context*: Documents must currently be used within Adult Social Care, or that guide ASC practice in England for Social Workers, Occupational Therapists and care providers or assessors. To obtain documents, organisations had to be contactable via email.

### Selection of sources of evidence

All returned documents were assessed for eligibility by two reviewers independently (LF and TK). Eligibility assessment took part in two stages. Documents were initially screened by title to remove obviously irrelevant documents and then by a full text read to determine eligibility. Agreement between reviewers was substantial (89%, Cohen’s *k*: 0.77). Any disagreements were resolved via discussion between LF and TK with a third reviewer used if necessary.

### Data charting process

Google Sheets was used to chart and extract data from the content of the provided documents and PRT tools to identify their consistencies, conflicts and/or differences. The extraction sheet was piloted and after finalised, 20% of the documents were independently extracted by JC and LN. If any discrepancies were revealed, a resolution was reached through a discussion between LF, JC and LN.

### Data items

The main items extracted from the data were as follows: author, type, inclusion of a tool, intended professional group, intended client group, the definition of risk, the definition of PRT, how the documents guide professionals, grading of risk, underpinning evidence and underpinning legislation.

### Synthesis

The Appraisal of Guidelines for Research and Evaluation II (AGREE II) instrument (AGREE Next Steps Consortium, 2017) was used to evaluate the quality of the documents included in this review. Appraisal domains included items such as ‘stakeholder involvement’ and ‘rigour of development’. For the specific content of PRT within the guidelines, the Template for Intervention Description and Replication Checklist (TIDieR; Hoffmann et al., 2014) was used as a framework to describe and synthesise PRT as an intervention. Specific data were characterised according to what, who provides, how and where. In this context, the tailoring item was interpreted as the guideline to ascertain what the PRT was for. The definitions for ‘risk’ and ‘PRT’ used in the documents were coded to identify the key concepts used to define these terms. Terms and methods used to describe the level of risk were also contrasted between documents. Document reference lists were synthesised to determine the most commonly cited sources and use of any evidence base. Given the nature of the documents included in this review, critical appraisal of the evidence was not undertaken but items within the documents were discussed.

## Findings

### Selection of sources of evidence

**Figure 1.**
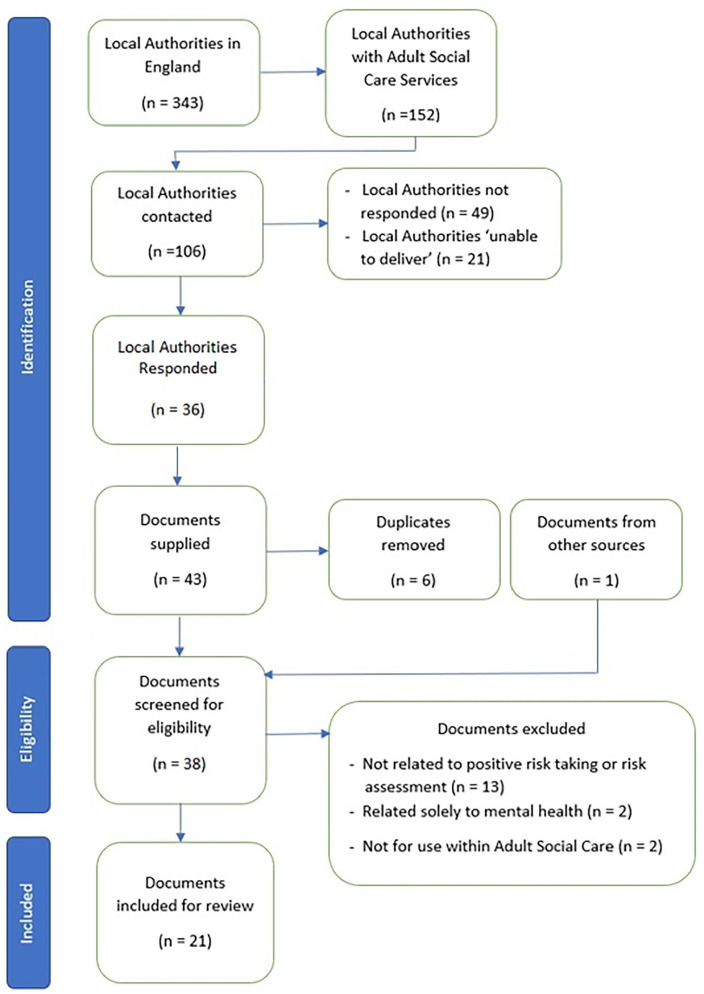
PRISMA flow diagram.

The PRISMA flow diagram is found in Figure 1. A list of 343 Local Authorities within England in 2019, available annually from The Ministry of Housing, Communities and Local Government, was utilised and 152 were identified from The National Audit Office as providing ASC services. Of those, 106 were contactable via email and contacted in October 2020. In all, 36 Local Authorities responded. In total, 21 email contact attempts were undelivered/rejected and 49 Local Authorities did not respond.

Of the 36 Local Authorities that responded, 22 Local Authorities provided a total of 43 documents that were screened against the eligibility criteria and one additional framework retrieved after examining grey literature. After duplicates were removed and eligibility was determined, a total of 21 documents were included for review (Table 1 for source documents). These consisted of five tools (for completion by professionals) and 16 documents (guidance for professionals). Within those 16 documents, a further eight tools were included either as appendices or at the end of the provided document.

**Table 1.** Sources of evidence.

Document, reference	Published by/date	Type	Overall AGREE II score	Risk Assessment included	Total page versus pages that mention PRT	Professionals intended for	Client group intended for
1. Warwickshire County Council (2020b)	Local Authority 2020	Guidance	3	Yes	12:0	ASC staff	ASC customers
2. Warwickshire County Council (2020a)	Local Authority 2020	Framework	2	No	11:2	ASC staff	ASC customers
3. East Sussex County Council (2015)	Local Authority 2015	Policy	2	No	6:0	ASC staff and health staff	ASC and mental health customers
4. East Sussex County Council (2017)	Local Authority 2017	Instructions	5	Yes	21:0	ASC staff	ASC customers
5. Kirklees Council (2013)	Local Authority 2013	Framework	3	No	11:3	ASC staff	ASC customers
6. Kirklees Council (2012)	Local Authority 2012	Guidance	4	Yes	14:7	ASC staff	ASC customers
7. Islington Council (2019)	Local Authority 2019	Framework	5	No	13:0	Professionals working with adults	ASC customers
8. Hampshire County Council (2019)	Local Authority 2019	Policy	5	Yes	54:10	ASC staff	ASC customers
9. Royal College of Occupational Therapists (2018)	Professional body 2018 (Provided by Medway Council and Derby City Council)	Guidance	5	No	28:28	OTs	Adults
10. Middlesborough Borough Council (2019)	Local Authority and Health service 2019	Guidance	4	Yes	37:2	OTs	ASC and health customers
11. Kent County Council (2020)	Local Authority 2020	Policy/guidance	5	Yes	36:7	ASC staff	ASC, transitions, learning disabilities
12. West of Berkshire Safeguarding Adults Board (2020)	Safeguarding Adults Board 2020 (Provided by Wokingham Borough Council)	Framework/guidance	5	Yes	24:9	ASC staff	ASC customers
13. Bournemouth, Christchurch and Poole Council (n.d)	Local Authority date not known	Tool	N/A	N/A	4:0	ASC staff	ASC customers
14. Calderdale Council (Not Known)	Local Authority date not known	Tool	N/A	N/A	2:0	ASC staff	ASC customers
15. South Gloucestershire Council (n.d)	Local Authority date not known	Tool	N/A	N/A	4:0	ASC staff	ASC customers
16. Bury Council (Not Known)	Local Authority date not known	Tool	N/A	N/A	16:0	ASC staff	ASC customers
17. Royal Borough of Windsor and Maidenhead (n.d)	Local Authority date not known	Tool	N/A	N/A	8:0	ASC staff	ASC customers
18. Positive Futures (2016)	Independent 2016	Framework	2	No	12:6	ASC management	ASC staff
19. Cornwall and Isles of Scilly Adult Safeguarding Board (2017)	Local Authority 2017 (Provided by Cornwall Council)	Policy	4	Yes	159:5	ASC staff	ASC customers
20. North Lincolnshire Safeguarding Adults Boardl (2020)	Local Authority 2020 (Provided by North Lincolnshire Council)	Policy	4	No	95:1	ASC staff	ASC customers
21. Gloucestershire Safeguarding Adults Board (2020)	County Council 2020 (Provided by Gloucestershire County Council)	Policy	4	No	135:4	ASC staff	ASC customers

NB: Document nine (RCOT, 2018) uses the term PRT four times, but prefers the term ‘positive risk enablement’ or ‘engaging with risk’ on every page.

### Reasons for not providing documents

Eight Local Authorities gave explicit reasons for not providing documents. Four reported that they did not use a tool or framework for PRT, two reported using external guidance – both used RCOT guidance (2018), and one reported they were looking to develop one in the near future. After clarification, one Local Authority reported that PRT is not a term they use and referenced a generic safeguarding policy.

### Document characteristics

#### Characteristics of sources of evidence

Table 2 shows an extract of the results of individual sources of evidence and that the approach to PRT within ASC has a face-to-face method of delivery. A strengths-based approach, which is to identify and utilise a person’s strengths and assets, is adopted within all documents by including the individual in the decision-making process and there was a consensus that risk assessments need to have action plans and be regularly reviewed.

**Table 2.** Extract of table informing synthesis of guidelines based on items from TIDieR.

	Brief name	Why	What – materials	What – procedures	How	Where	When and how much	Tailoring
1	Risk Assessment	To enable a person to make a supported decision whilst maintaining their autonomy. To consider risk and mitigation to as best as possible maintain a person’s choice and safety.	Tool and guidance document	Specific step-by-step guidance on completing an electronic risk assessment.	Face to face	Within an Adult Social Care setting – that is, the client’s home or in the community	When there is concern regarding a person’s choice and associated risk – and they have care and support needs. There is an option at the end for ‘review of risk assessment’ but no indication of when or how often this should be completed.	In this context, the tailoring item was interpreted as the guideline to ascertain what the positive risk-taking was for.
2	Risk Management Framework – A Guide	To enable a person to make a supported decision whilst maintaining their autonomy. To consider risk and mitigation to as best as possible maintain a person’s choice and safety.	Guidance document only	Strongly considers strengths-based practice and considering of multi-agency working when completing the assessment with the customer.	Face to face	Within an Adult Social Care setting – that is, the client’s home or in the community	When there is concern regarding a person’s choice and associated risk – and they have care and support needs. There is an indication that practitioners should agree on a date for review but no indication of when this should be or how often this should be reviewed.	In this context, the tailoring item was interpreted as the guideline to ascertain what the positive risk-taking was for.

AGREE II appraisal found all guidelines had clear objectives and were specific to whom the guidelines were meant to apply (Scope and Purpose: median 7 (IQR 7–7)). Views and preferences of service users appeared absent in all documents and the median Stakeholder Involvement domain score was 4 (IQR 2–4). The lowest domain scores found were for Rigor of Development (median 2 (IQR 1–2)) and Editorial Independence (median 1 (IQR 1–2)). Overall, 10 documents were assessed as not recommended and 6 recommended with modifications. AGREE II scores were unable to be determined from 5 documents due to them being stand-alone tools rather than guidelines.

The tools provided varied dramatically in their length (from 2 to 16 pages), information-gathering requirements and terminology. This was largely due to the type of tool provided. Ten tools (Source documents 1, 4, 8, 10, 11, 12, 13, 14, 15 and 17) were designed for the assessment of a specific risk and therefore were more concise as there was a specific aim, whilst others were more generic risk assessments looking at the person’s life as a whole, rather than a specific activity. Two documents (6 and 16) were full social care assessments that incorporated all domains of The Care Act and therefore were longer in length. One document was structured as a full safeguarding enquiry (19), which would usually be completed following a concern of harm occurrence or potential, rather than because of a person choosing/wishing to participate in PRT.

In all, 13 documents instruct professionals to complete a risk assessment document (Table 1). Four documents (12, 19, 20 and 21) provide a flow chart of the organisation’s steps of risk management or safeguarding processes, while three documents (9, 10 and 11) provide a diagrammatic representation of the risk assessment process to guide and structure the professional’s clinical reasoning. Three documents (1, 2 and 7) specifically guide the professional to consider strengths-based practice, and three documents (2, 7 and 12) ask the professional to consider multi-agency working.

Another document (20) guides professionals to structure their clinical reasoning according to local risk management stages, whilst a further document (9) instructs professionals to document their assessment according to the Health and Care Professions Council and RCOT standards of practice.

#### Definition of the term ‘risk’

Nine documents (3, 5, 8, 9, 10, 11, 12, 20 and 21) gave their definitions of the term ‘risk’ – some adapted from other sources. The synthesis of which elicited three main concepts that were the essence of their definitions in this context. These were that risk is not a certainty, but the likelihood of a hazard occurring; clarity that the outcome of the risk-taking could be both positive and negative; and that the consequences could affect both the individual taking the risk and others around them. Although there was no consistent definition of risk in the context of PRT, the severity of harm was also considered within three document definitions (3, 9 and 11).

#### Definition of the term ‘Positive Risk Taking’

There was no consistent definition of the term ‘Positive Risk Taking’. Nine documents (5, 9, 8, 10, 11, 12, 18, 19 and 21) defined PRT, with three documents citing sources (8, 10 and 11). The documents identified that PRT is a process of identifying the potential risks of an individual making a decision/participating in an activity, and weighing those risks against the potential benefits to the individual. Other main concepts were that PRT is to enable an individual to exercise their choice and autonomy and that the PRT process includes setting clear goals to be achieved. Four documents (8, 5, 10 and 19) were clear that a plan and actions must be developed for mitigation of the identified risks. One document incorrectly used the term ‘positive risk’ twice within their document by defining the risk itself as positive, rather than the outcome (6).

#### Risk assessment

Five documents provided case examples within their text or as partly completed tools (1, 8, 9, 10 and 21). However, one was not explicitly for PRT, but an example of a safeguarding concern (21). Two provided example scenarios around manual handling and accommodation arrangements and applied these to PRT (1 and 10). Case examples in Document 9 were tailored to guide a professional’s thinking towards the potential negative outcomes of not embracing and engaging with risk for individuals.

Of the 21 documents provided, 12 documents did not require the level of risk to be graded (1, 2, 5, 7, 8, 9, 10, 13, 15, 16, 18 and 21). However, of those 12, five documents suggested that professionals should be assessing the level of risk according to the likelihood of the risk occurring and the severity of the risk occurring (1, 9, 10, 16 and 21), but no guidance was provided to aid the professional in determining the level or grade of risk. Nine documents did require the grading of risk to be recorded (3, 4, 6, 11, 12, 14, 17, 19 and 20). Of those nine, six documents specifically guided professionals by providing a risk matrix (3, 4, 6, 11, 19 and 20); however, none of the matrices were consistent with their terminology. For example, the descriptor ‘minor’ was used at three different stages of grading between documents (Table 3).

**Table 3.** Terminology used to describe levels of risk.

	Level of risk				
Descriptor	Negligible	Low	Minor	Significant	Severe
	Negligible	Minor	Moderate	Major	Catastrophic
	Minor	Moderate	Significant	Serious	Major
	Low		Medium		High
	Minor	Moderate	Significant	Very Significant	Critical

Eighteen of the 21 documents did indicate that risk assessments should be reviewed and three did not (6, 16 and 18). Of those three, one document (6) was unclear in indicating that a review would be undertaken by providing a free-text box within a tool, titled ‘*Monitoring – By Whom*’. Of those that indicated a review was required, only one stated a timeline of *‘at least annually’* (4), and Document 6 provided options for review at 3, 6 and 12 months depending on the grading of the risk. There appeared to be no guidance in any other document for when to set a review date.

#### Cited sources within documents

In all, 15 documents provided for the review referenced legislation (1, 2, 3, 4, 5, 6, 7, 8, 9, 10, 11, 12, 19, 20 and 21). The most referenced legislations were as follows:

- 12 × citations of The Mental Capacity Act 2005
- 8 × citations of The Care Act 2014
- 7 × citations of The Human Rights Act 1998
- 4 × citations of The Data Protection Act 1998
- 4 × citations of The Equality Act 2010
- 3 × citations of The Mental Health Act 1983
- 2 × citations of the Health and Social Care Act 2012
- 2 × citations of General Data Protection Regulations 2018

The six documents that did not reference any legislation were five standalone tools (13, 14, 15, 16 and 17) and one framework which was intended for social care management (18).

Eight documents cited references that were not legislation to inform the guidelines (5, 8, 9, 10, 11, 12, 18 and 21). One document (18) cited sources but did not include a reference list and one document (12) stated that it had been produced/adapted from four Local Authority sources. Three documents specifically cited the Department of Health’s six principles of safeguarding (19, 20 and 21) to aid professionals in structuring their clinical reasoning.

#### Addressing barriers to PRT

Three documents (3, 9 and 18) acknowledged a fear of repercussions or fear of a punitive culture within professionals, regarding PRT with adults, that discourages their engagement. A risk-averse culture was identified in that:

Those who use social care services frequently report that risk taking is discouraged, either because of perceived limitation or fear that they or others might be harmed. In this instance, risk was regarded solely in negative terms and there was little thought given to the positive experiences to be gained from taking risks. (18)

Document 18 attributed their source to Glasby (2011). A further two documents concurred by also acknowledging the consequences of not taking the risk – loss of autonomy/restriction of choice for the individual concerned (2 and 9). Two documents (6 and 18) made specific statements concerning training for professionals and the application of PRT. Document 6 stated:

PRT should be ingrained into the working culture and be reflected in the context of team training. It is not a one-off experiment but the natural first line of thinking. (6)

## Discussion and implications

This review found a large variation in the guidance documents for professionals to support their clients to engage in PRT within ASC in England. Notably, there was little consensus on the definitions of risk and PRT and how to operationalise risk assessment to support risk enablement for adults with a physical disability.

Of the 106 Local Authorities contacted, only 18 provided eligible documents (Table 1). The reasons given for not providing any documents were largely due to either not having any guidance in place or being unfamiliar with the term PRT. This further highlights the inconsistency of PRT provision and support across England.

However, consistencies exist across the documents used for PRT. The results of this review highlight that all known documents are intended to be used face to face with the individual and with a strengths-based approach. We also found that Local Authorities use risk assessment matrices to determine levels of risk – though the matrices differed, as did the terminology used. This has also been observed in the National Health Service (NHS), where different matrices are used – for example, for violence against employees and prevention of pressure ulcers (Moore and Patton, 2019).

Although the term ‘risk’ does not automatically denote a positive or negative outcome, many resources including published dictionaries include within their definition of risk, the likelihood of a harmful outcome. These negative connotations with the term ‘risk’ often lead professionals to associate risk with harm and negative outcomes. Morgan (2010) states that evidence ‘*in the context of risk is entirely negative*’ – in that research is conducted into what has and could go wrong. Research is required to determine practices that are working well for PRT.

Whilst similar concepts were found throughout definitions of PRT, we found no consistently used definition. Inconsistency in definitions impedes the full understanding of the practice, and terminology needs to be further explored to provide a consensus for the terminology used. The variation we observed in the terminology used may lead to misinterpretation of the level of risk across localities.

From synthesising the existing definitions of Positive Risk Taking, the concepts mostly included are that PRT is the identification of positive outcomes for the individual, as well as the potential harms stemming from the risk being taken, and weighing those up to make an informed decision. Definitions also stated the need for the inclusion of an action plan to mitigate the risk, for the individual to achieve their goals and exercise their choice and autonomy. Having clear and consistent policies and procedures, and approaches that are shared and understood by everyone are considered good standards of practice by the RCOT.

The two documents (6 and 16) that guided professionals to complete a full care needs assessment were lengthy. Whereas those that were designed to assess the risks associated with a specific decision were more concise in the information they were required to capture, as well as shorter in length. Although there is no known formal recommendation for minimum or maximum length of a document, the National Office of Statistics states that shorter documents are preferable to increase engagement with the text.

A feature of the shorter tools we found within our review was that they also tended to provide more free-text boxes for recording information in addition to tick boxes. This is consistent with the recommendations of Oppenheim (1992) of incorporating a mix of these recording methods to allow for both depth and personalisation of information recording and a structure that is not open to misinterpretation. The Risk Matrix Approach (Ni et al., 2010) provides a visual semi-quantitative risk assessment to complement a PRT tool.

Further to the tools, only two documents (4 and 6) specified a risk assessment review timeline. These documents recommended an annual review which is consistent with the Health and Safety Executive recommendation. The National Archive recommendation for a full risk assessment review is at least every 2 years or upon a change in circumstances. Having specific criteria for the frequency of reviews may further support a standardised approach to PRT and should be considered in future research.

We only found two documents that included tangible examples of PRT within their guidance (1 and 8). Tangible examples have been identified as an important factor to support professional confidence in applying PRT to practice (Seale et al., 2013). While the extent to which professionals are trained in using the guidelines could not be determined from our review, providing real-life scenarios of complex cases within training materials could ‘*stimulate discussion and challenge individual viewpoints*’ (Positive Futures, 2016). As the tools are designed for use by ASC professionals, providing training to this population may help to address concerns of a lack of senior support in this area of practice and address the PRT barriers of ‘*No Support*’ and ‘*Blame Culture*’ as identified by Newman et al., (2022b).

The AGREE II instrument evidenced that most documents did not reference literature to support the guidance beyond legislation. An exception to this was the RCOT document which included five pieces of research literature. However, the literature cited was from expert opinion, and/or relating to mental health practice. This suggests there is a paucity of direct evidence to inform PRT for adults with physical disability. In addition, service user involvement in the development of guidelines could inform a more strengths-based approach to PRT.

The six principles of safeguarding are referred to throughout the literature and within three documents (19, 20 and 21), to support professionals in promoting effective service user involvement in making safeguarding personal (Droy and Lawson, 2017). Given the limited direct evidence and subjective nature of assigning value to potential benefit, these principles seem appropriate to consider for PRT and should be familiar to social care professionals, as they have been incorporated into The Care Act. These principles structure a professional’s initial clinical thinking, to then be considered in conjunction with more tailored guidelines for PRT.

Specific recommendations for PRT are provided in Morgan (2004), cited in three documents (8, 10 and 12). Although this report evolved from Morgan’s work within mental health practice, their guidelines were successfully transferred to the documents developed by the Local Authorities that cited this report. By contrast, guidelines for PRT within mental health practice have a larger evidence base to support recommendations (Southern Health NHS Foundation Trust, 2012). A formal review of the evidence is required to determine the extent of the evidence base in physical health practice.

Based on the synthesis of the existing guidance to professionals for completing a PRT risk assessment, some core elements could be considered to standardise an approach and inform a most practical and complete guide or tool for PRT. These are the inclusion of instructions for completing/recording risk assessment within a pro-forma document, as well as guiding the professional’s clinical reasoning by highlighting the six principles of adult safeguarding. The inclusion of a risk matrix tool is useful in grading the level of risk and guidelines should include instructions for how to utilise a matrix, as well as guidelines to determine the timeframe for reviewing according to each individual. Although few documents specifically guided professionals to think about multi-agency working and a strengths-based approach (though tool designs indicated a strengths-based approach), this is also important for assessment transparency, sharing or gathering necessary information, and certainly for keeping the person at the centre of all discussions.

### Strengths and limitations

To our knowledge, this is the first review to systematically examine PRT guidance documentation.

**Figure 2.**
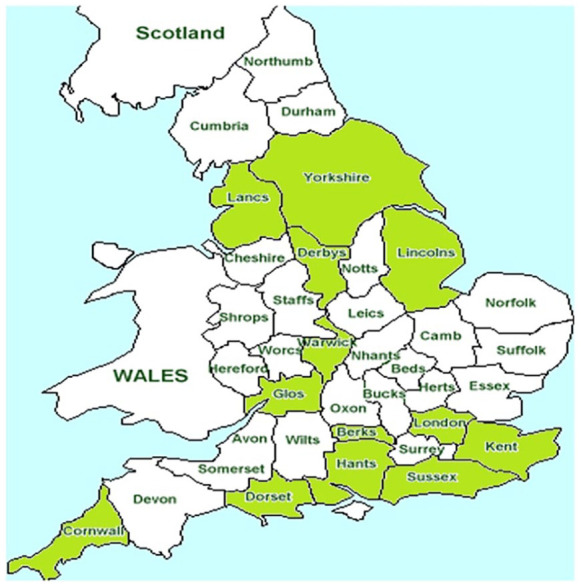
Visual representation of returned documents.

Although 70% of Local Authorities in England with ASC services were able to be contacted via valid email addresses (106 out of 152), not all responded (36 out of 106). It should be considered that this project was completed during the pandemic which could have contributed to non-response. We had documents returned from Local Authorities across England, and whilst Figure 2 shows a broad geographical representation across the country, it is important to consider that there are limitations in the generalisability of our findings. However, here we present insight that may be useful for those enabling PRT to consider within their context.

Where further documents were cited within guidelines, the reference lists of those documents were not fully explored. Lastly, a 100% second review of the extracted data items was not available, though a 20% review was completed with excellent agreement.

## Conclusion

A variety of documents for PRT are in use across ASC services in England. The results of this review could help guide future document development and the inconsistencies identified support the adoption of a more standardised and unified approach. Particularly, the current inconsistencies in grading risk and terminology used could have implications in practice by leading to misinterpretation and potentially affect the level of any mitigation required – indeed any safety measures put in place to mitigate risk may not be adequate if the risk level has been misunderstood.

The extent to which social care professionals are trained in the practice of PRT is unknown and education on the use of risk assessment tools has been identified as a need. Further research into professionals’ training could elicit the enablers to engaging with PRT. It is also unclear if there has been any professional/service user input into the design of the guidelines currently in use.

There appears to be a paucity of specific empirical evidence to inform guidelines, which has likely contributed to the varying approaches to PRT. Thus, there is a need to identify the nature and extent of the current evidence for PRT in the context of adults with physical disabilities, to inform areas of future inquiry and inform an evidence-based application of Positive Risk Taking, to support professionals’ confidence and widen access to individuals who may benefit from this practice.

**Key findings**

- A strengths-based approach to PRT
- An inconsistency with the terminology used and grading of risk
- There appears to be a paucity of direct evidence to inform PRT guidelines

**What the study has added**

This is the first specific summary of guidelines used across Local Authorities in England and identifies gaps in the evidence base which informs areas of further inquiry, leading to establishing an evidence-based application of PRT.
